# Prediction of 12-Week Remission in Patients With Depressive Disorder Using Reasoning-Based Large Language Models: Model Development and Validation Study

**DOI:** 10.2196/83352

**Published:** 2026-01-23

**Authors:** Jin-Hyun Park, Hee-Ju Kang, Ji Hyeon Jeon, Sung-Gil Kang, Ju-Wan Kim, Jae-Min Kim, Hwamin Lee

**Affiliations:** 1 Department of Biomedical Informatics, Korea University College of Medicine, 161, Jeongneung-ro, Seongbuk-gu, Seoul, 02708, Republic of Korea, 82 2-3407-2099; 2 Department of Psychiatry, Chonnam National University Medical School, Gwangju, Republic of Korea

**Keywords:** artificial intelligence, clinical support systems, depressive disorder, large language models, natural language processing, prognosis, treatment outcome

## Abstract

**Background:**

Depressive disorder affects over 300 million people globally, with only 30% to 40% of patients achieving remission with initial antidepressant monotherapy. This low response rate highlights the critical need for digital mental health tools that can identify treatment response early in the clinical pathway.

**Objective:**

This study aimed to evaluate whether reasoning-based large language models (LLMs) could accurately predict 12-week remission in patients with depressive disorder undergoing antidepressant monotherapy and to assess the clinical validity and interpretability of model-generated rationales for integration into digital mental health workflows.

**Methods:**

We analyzed data from 390 patients in the MAKE Biomarker discovery study who were undergoing first-step antidepressant monotherapy with 12 different medications, including escitalopram, paroxetine, sertraline, duloxetine, venlafaxine, desvenlafaxine, milnacipran, mirtazapine, bupropion, vortioxetine, tianeptine, and trazodone, after excluding those with uncommon medications (n=9) or missing biomarker data (n=32). Three LLMs (ChatGPT o1, o3-mini, and Claude 3.7 Sonnet) were tested using advanced prompting strategies, including zero-shot chain-of-thought, atom-of-thoughts, and our novel referencing of deep research prompt. Model performance was evaluated using balanced accuracy, sensitivity, specificity, positive predictive value, and negative predictive value. Three psychiatrists independently assessed model outputs for clinical validity using 5-point Likert scales across multiple dimensions.

**Results:**

Claude 3.7 Sonnet with 32,000 reasoning tokens using the referencing of deep research prompt achieved the highest performance (balanced accuracy=0.6697, sensitivity=0.7183, and specificity=0.6210). Medication-specific analysis revealed negative predictive values of 0.75 or higher across major antidepressants, indicating particular utility in identifying likely nonresponders. Clinical evaluation by psychiatrists showed favorable mean ratings for correctness (4.3, SD 0.7), consistency (4.2, SD 0.8), specificity (4.2, SD 0.7), helpfulness (4.2, SD 1.0), and human likeness (3.6, SD 1.7) on 5-point scales.

**Conclusions:**

These findings demonstrate that reasoning-based LLMs, particularly when enhanced with research-informed prompting, show promise for predicting antidepressant response and could serve as interpretable adjunctive tools in depressive disorder treatment planning, although prospective validation in real-world clinical settings remains essential.

## Introduction

Depressive disorder is one of the most prevalent and debilitating psychiatric conditions worldwide, ranking as a primary contributor to global disability and significantly influencing the overall disease burden associated with mental disorders [1]. Given the substantial burden imposed by depressive disorder, optimizing strategies for early diagnosis, effective treatment, and personalized intervention remains a critical public health priority. Despite the critical need for effective intervention, the primary treatment objective of achieving remission, defined as near-complete symptom resolution, remains challenging, with initial antidepressant monotherapy resulting in remission rates of only 30% to 40% within 12 weeks [2,3]. This limited success often necessitates multiple treatment trials, consequently prolonging suffering, increasing health care use and suicide risk, elevating dropout rates [4], and ultimately exacerbating patient distress while significantly amplifying treatment nonadherence [5].

Consequently, the early identification of patients who will not achieve remission with a particular monotherapy regimen has become a critical topic in both research and clinical practice. Early identification of patients who are less likely to respond to standard first-line treatments would allow clinicians to tailor interventions more efficiently and reduce the time lost during ineffective treatments [6]. Recent studies have explored the use of machine learning (ML) models to predict remission in patients with depressive disorder. However, these investigations have encountered limitations, resulting from study design, which may not reflect real-world clinical practice, including limited diversity in the antidepressants administered and challenges in clinically interpreting the predictions generated by ML models [7-10].

In recent developments, large language models (LLMs) have emerged as promising instruments for various psychiatric applications, encompassing diagnostic assessment, risk stratification, and clinical decision support [11-13]. Furthermore, LLMs that enhance chain-of-thought reasoning, such as OpenAI’s ChatGPT o1 [14], ChatGPT o3-mini [15], and Anthropic’s Claude 3.7 Sonnet [16], have been developed and applied within the medical field to improve diagnostic reasoning. These reasoning-enhanced LLMs have demonstrated potential across various medical specialties, yet their application to predicting antidepressant treatment outcomes remains unexplored [17-21].

Therefore, in this study, we aimed to evaluate whether reasoning-enhanced LLMs could accurately predict 12-week remission among patients with depressive disorder undergoing monotherapy with 1 of 12 different antidepressants, including selective serotonin reuptake inhibitors (SSRIs), serotonin and norepinephrine reuptake inhibitors (SNRIs), or other antidepressants. We also investigated the underlying clinical rationale of these predictions and explored the feasibility of proposing alternative treatment strategies when remission was deemed unlikely.

## Methods

### Participants and Data Preprocessing

The dataset for this study was obtained from the MAKE Biomarker Discovery for Enhancing Antidepressant Treatment Effect and Response (MAKE BETTER) study [22]. Patients with depressive disorders were consecutively recruited from March 2012 to April 2017 at the outpatient psychiatry department of Chonnam National University Hospital. From the initial cohort, 431 patients who received first-step monotherapy were identified. After excluding 9 patients prescribed “other” medications and 32 lacking blood biomarker data, a total of 390 patients were included in the final analysis.

Variables assessed included demographic characteristics, personal and familial psychiatric histories, comorbidities, responses to the 9-item Mini-International Neuropsychiatric Interview [23], adverse childhood experiences before the age of 16 years (physical, psychological, and sexual abuse), depression subtypes (including melancholic, atypical, and psychotic), and prescribed antidepressants and dosage. Suicidality was assessed using a structured interview comprising 4 standardized questions addressing suicidal thoughts and intent (eg, *“*Have you ever felt that life is not worth living?*”*). The presence of suicidal ideation determined from these structured questions was subsequently reflected in the Brief Psychiatric Rating Scale [24] suicidality item rating. For analysis, only the binary presence or absence of suicidal ideation was used, not the raw Brief Psychiatric Rating Scale score. Additional variables included the Hamilton Depression Rating Scale (HAM-D) [25] score, health-related quality of life (EQ-5D) [26], functional impairment (Sheehan Disability Scale) [27], perceived stress (Perceived Stress Scale) [28], resilience (Conner-Davidson Resilience Scale) [29], perceived social support (Multidimensional Scale of Perceived Social Support) [30], blood biomarkers at baseline, and early treatment response at 2 weeks (≥20% reduction in HAM-D scores). For female participants, fertility and depression-related factors were evaluated, including age at menarche or menopause, hormonal therapy use, and presence of peri- or postpartum or postmenopausal depression. Further details on eligibility, pharmacotherapy, clinical assessments, and biomarker procedures are provided in Multimedia Appendix 1. The primary outcome was 12-week remission, defined as an HAM-D score ≤7 sustained through the 12-week assessment point. All analyzed participants were adults, consistent with the validated use of psychiatric assessment tools and pharmacotherapy in adult outpatient clinical practice.

Numeric coded data were transformed into structured, narrative-style reports in natural language to enhance interpretability by the LLMs, and the comprehensive structure of patient information is depicted in Textbox 1.

Textbox 1. **Structured representation of patient information used for input to the large language models (LLMs). This figure illustrates the structured format of patient information for individuals with major depressive disorder as prepared for LLM input. Each patient’s clinical data were inserted into the (patient information) section of the experimental prompt template for subsequent model evaluation.**

(Patient information)

(Basic information)
  - Age: xx years
  - Sex: Male or Female
  - Height: xxx.x kg
  - Weight: xx.x kg
  - Smoking status: Non-smoker, Ex-smoker or Current smoker
  - Drinking pattern: Non-drinker, E-drinker, or Current drinker
  - Alcohol Use Disorders Identification Test (AUDIT) score: (For patients who are current drinkers)
  (Female-specific information)
  - Childbearing potential: Yes or No
  - Pregnancy experience: Yes or No
  - Pregnancy during pregnancy: Yes or No
  - Postpartum depression syndrome: Yes or No
  - Age at menopause: xx years
  - Postmenopausal syndrome: Yes or No
  - Onset of depression at menopause: Yes or No
  (Comorbidities) (All applicable conditions, if any)
  - Allergic/Immunologic disease, Heart disease, Hypertension, Stroke, Respiratory disease, Dermatologic disease, ear , nose and throat (ENT) disease, Endocrine disease, Ophthalmic disease, Gastrointestinal disease, Genitourinary disease, Hematologic cancer, Solid tumor, Musculoskeletal disease, and/or Neurological/Parkinson disease
  (Depression subtype) (All applicable conditions, if any)
  - Anxious, Melancholic, Atypical, or Psychotic
  (Monotherapy and 2-week Response)
  - Main AD (12w): Escitalopram, Paroxetine, Sertraline, Duloxetine, Venlafaxine, Desvenlafaxine, Milnacipran, Miratazapine, Bupropion, Vortioxetine, Tianeptine, or Trazodone
  - Mean dose (12w): xx.x mg - ADT equivalent dose: (12 w): xx.xxx mg
  - Early response at 2 wells (≥20% HAM-D decrease): Yes or No
  (Social-psychological assessments)
  - HAM-D (Hamilton Depression Rating Scale) total score: xx
  - EQ-5D (EuroQol-5 Dimension) index: x.xx
  - SDS (Sheehan Disability Scale) total score: xx
  - PSS (Perceived Stress Scale) total score: xx
  - CD-RISC (Connor-Davidson Resilience Scale) total score: xx
  - MSPSS (Multidimensional Scale of Perceived Social Support) average score: x.xxx
  (Biomarkers)
  - High-sensitivity C-reactive protein (hs-CRP): xxx mg L
  - Tumor necrosis factor-alpha (TNF-α): xx.xx pg/mL
  - Interleukin- 1 beta (IL-1ß): x.xx pg/mL
  - Interleukin-6 (IL-6): x.xxx pg/mL
  - Interleukin-4 receptor (I-4R): xxxxx pg/mL
  - Interleukin-10 (I-10): xxxxx pg/mL
  - Leptin: xx.xx ng/mL
  - Ghrelin: xxxxx pg/mL
  - Total Cholesterol: xxx mg/dL
  - Brain-derived neurotrophic factor (BDNF): xxxx ng/mL
  (Mini-International Neuropsychiatric Interview: MINI) (Yes or No)
  - Over the past 2 weeks, have you felt depressed or down most of the day, nearly every day?
  - Over the past 2 weeks, have you experienced a significantly decreased interest or pleasure in most activities or things you usually enjoy?
  - Have you had a nearly daily decrease or increase in appetite, or an unintentional weight loss or gain (±5% of your body weight in 1 month)? If either is Yes, record Yes.
  - Have you had insomnia or hypersomnia nearly every day (difficulty falling asleep, trouble staying asleep, early morning awakening, or sleeping too much)?
  - Have you spoken or moved more slowly than usual, or have you felt restless or unable to sit still nearly every day? If either is Yes, record Yes.
  - Have you felt fatigue or loss of energy nearly every day?
  - Have you felt worthless or guilty nearly every day?
  - Have you had difficulty concentrating or making decisions nearly every day?
  - Have you had recurrent thoughts of self-harm, suicidal ideation, or a wish for death?

### Ethical Considerations

This study was approved by the Chonnam National University Hospital Institutional Review Board (CNUH 2012‐014). Written informed consent was obtained from all participants. For minors, parental permission and child assent would have been required under institutional and national regulations; however, no minors were enrolled in this study.

### Study Design and Zero-Shot Prompting

**Figure 1.**
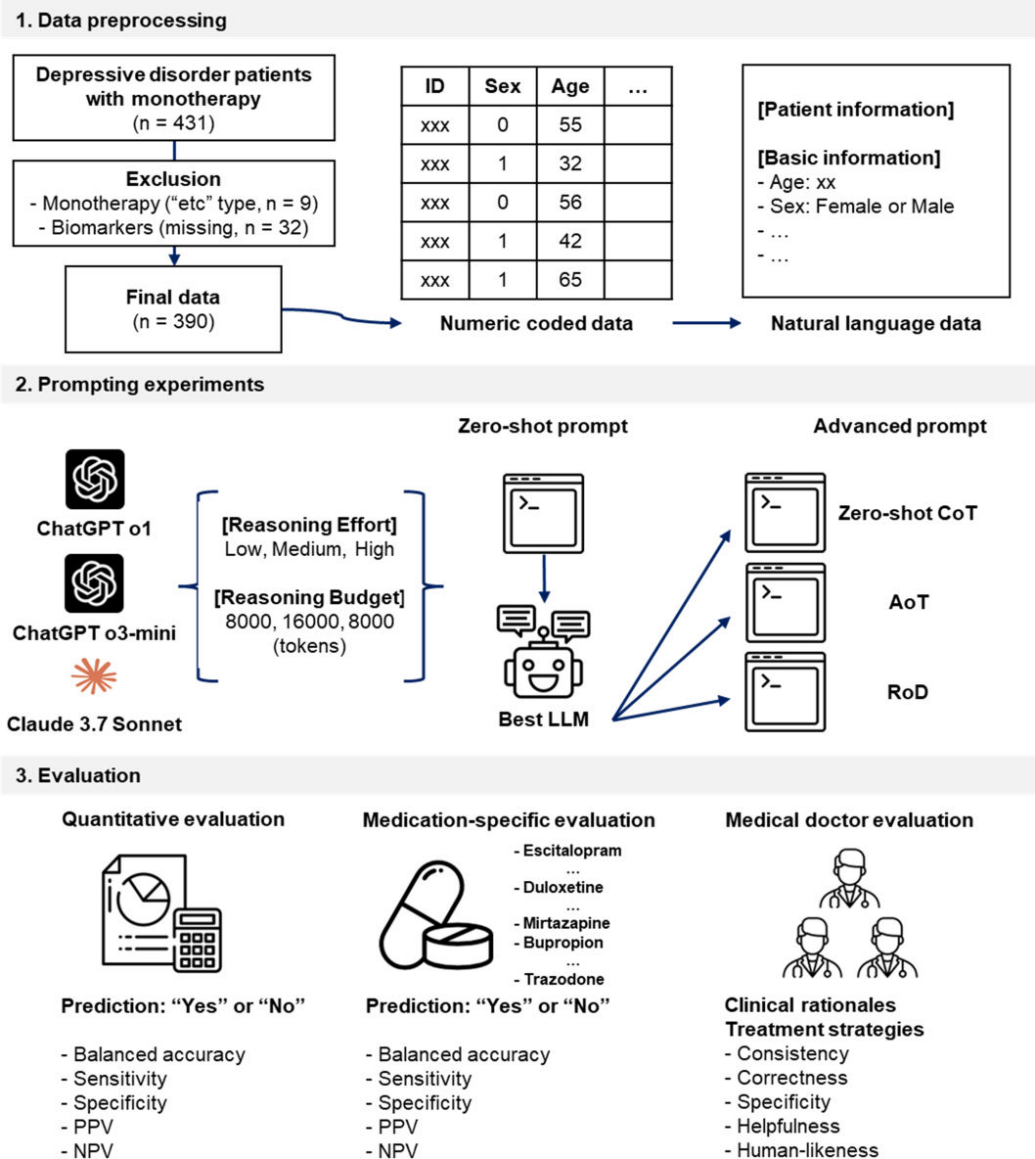
Methodological framework for LLM-based prediction of 12-wk remission in patients with depressive disorder. This figure depicts the three-phase methodological approach used in this study: (1) data preprocessing of depressive disorder patients with monotherapy (n=390), including transformation from numeric coded data to natural language format; (2) prompting experiment design; and (3) a comprehensive evaluation framework encompassing quantitative, medication-specific, and clinical assessments. AoT: atom-of-thoughts; CoT: chain-of-thoughts; LLM: large language model; NPV: negative predictive value; PPV: positive predictive value; RoD: referencing of deep research.

This study follows the Transparent Reporting of a multivariable prediction model for Individual Prognosis or Diagnosis guidelines. The design flow is illustrated in Figure 1. Initially, we conducted data preprocessing to prepare input for the LLMs. Subsequently, we used 3 reasoning-based LLMs, including ChatGPT o1 and o3-mini (OpenAI) and Claude 3.7 Sonnet (Anthropic), via an application programming interface to predict 12-week remission in patients with depressive disorder, generating clinical rationales for each prediction and treatment strategies for patients anticipated to not achieve remission; each output consisted of 5 distinct sentences.

We conducted zero-shot experiments to assess the performance of these LLMs. OpenAI’s models were evaluated across 3 levels of “reasoning effort” parameters (low, medium, and high), while the Anthropic model was tested at 3 reasoning budget token settings (8000; 16,000; and 32,000 tokens). The detailed structure of the zero-shot prompt is illustrated in Textbox 2.

Textbox 2. **Structure of zero-shot prompt. The prompt message remained consistent across all experiments, with only the (patient information) section being systematically replaced with individual patient data for each experimental case.**

(Zero-shot prompt)

You are an experienced psychiatrist specializing in depressive disorder. You can access a depressive disorder patient's baseline data, including monotherapy prescribing information and 2-week response.

Your task:

1. Predict the depressive disorder patient's 12-week remission as “Yes” or “No.”
2. Provide a “Clinical Rationale” of exactly five sentences (1~5).
3. If you predict “No,” also provide the next “Treatment Strategy” of exactly five sentences (1~5).
4. Final Output Format (follow precisely):

Remission prediction <Yes or No>

Clinical Rationale:

1. ...
2. ...
3. ...
4. ...
5. ...

Treatment Strategy (only if you predict “No”)

1. ...
2. ...
3. ...
4. ...
5. ...

Below is the patient's baseline data, including (Basic Information), (including (Female-specific Information) if the patient is female), (Comorbidities), (Mini-International Neuropsychiatric Interview), (Depression Subtype) if present, (Adverse Childhood Experiences (ACEs)) if present, (Depression History & Suicidality), (Monotherapy & 2-week Response), (Social-Psychological Assessments), and (Biomarkers).

Please use this data to predict the 12-week remission status (Yes/No) and follow the instructions above.

(Patient Information):

The best-performing zero-shot model, based on balanced accuracy, was further evaluated using advanced prompting strategies to enhance reasoning and interpretability. Specifically, the zero-shot chain-of-thought (CoT) prompting method [31] and the atom-of-thoughts (AoT) technique [32], both of which have shown strong performance on benchmark datasets, were adapted for this study. We also introduced a novel “referencing of deep research (RoD)” prompting strategy, which leverages OpenAI’s deep research [33] to generate research reports that are subsequently incorporated into the zero-shot prompt for additional context.

Finally, our evaluation process comprised multiple sequential phases. First, we conducted a comprehensive quantitative assessment of the zero-shot prompting approaches. Subsequently, using the best-performing model identified through this initial evaluation, we implemented the advanced prompting experiments and subjected them to identical quantitative evaluation methodologies. For the best advanced prompting model, we then performed medication-specific evaluations. Additionally, board-certified medical doctors evaluated the model-generated rationales and treatment strategies.

### Advanced Prompting

The zero-shot CoT was implemented by inserting the phrase “Let’s think step by step” immediately before the patient information section in the original zero-shot prompt.

The AoT technique comprised three phases: decomposition (breaking the question into subquestions), contraction (consolidating into an “atomic” question), and final solve (generating predictions with rationale, and, if necessary, subsequent treatment strategies). Detailed prompts appear in Multimedia Appendix 2.

**Figure 2.**
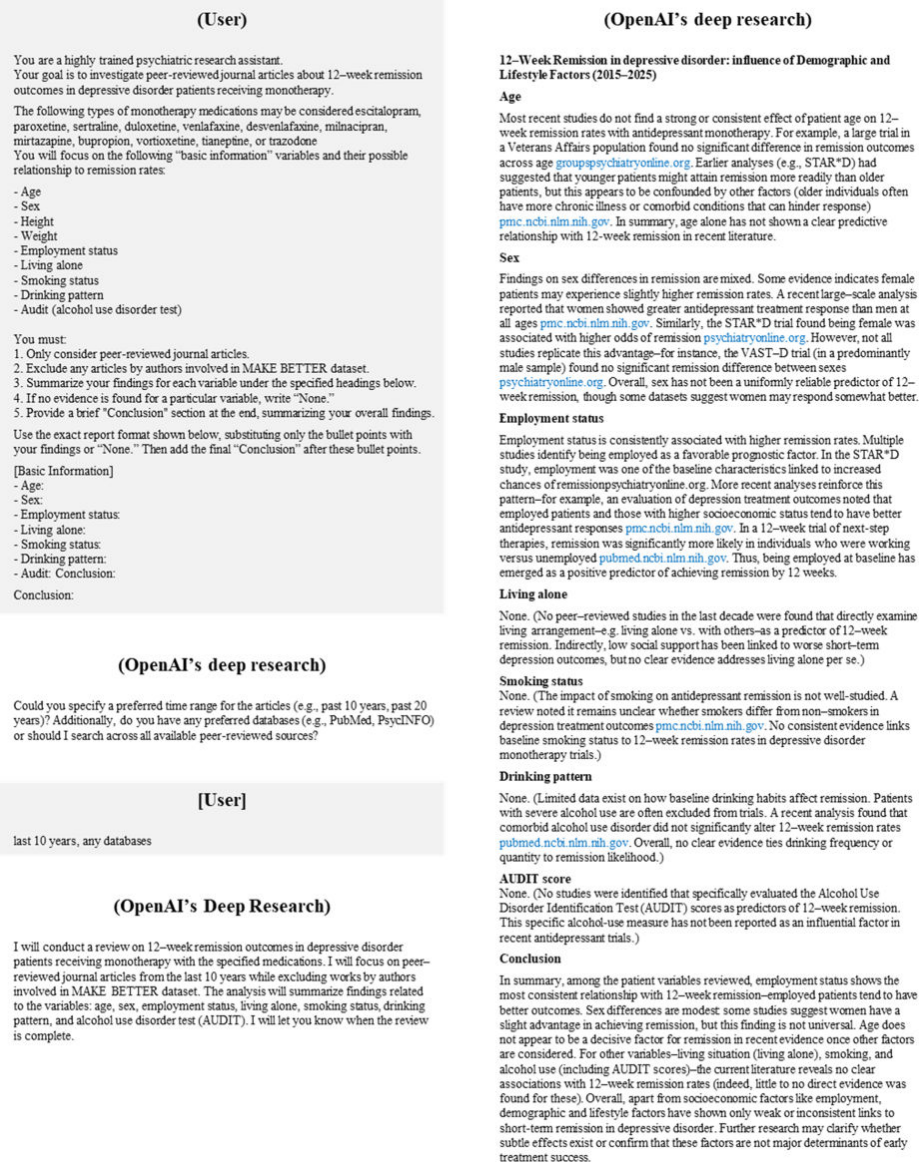
A representative dialogue example of “deep research report” generation. This figure illustrates an example dialogue where the user uses OpenAI’s deep research functionality to generate a “deep research report.” MAKE BETTER study: MAKE Biomarker Discovery for Enhancing Antidepressant Treatment Effect and Response study.

Furthermore, we introduced an RoD prompt to incorporate external evidence regarding 12-week remission outcomes. First, we used OpenAI’s deep research [33] to investigate prior studies on the relationship between our variables of interest and 12-week remission outcomes. A representative dialogue example demonstrating the generation of “deep research report” is presented in Figure 2.

This systematic examination deliberately excluded publications authored by contributors to the MAKE BETTER dataset to mitigate potential confirmation bias and ensure methodological independence in our analysis framework. We then consolidated these findings into a “deep research report” and subsequently integrated this report into the zero-shot prompt to design the RoD prompt. The prompt for conducting the “deep research report” and the RoD prompt is presented in Textbox 3.

Textbox 3. **The prompts of deep research and referencing of deep research. The deep research prompt template, used for OpenAI’s deep research functionality, instructs the model to function as a research assistant. The hierarchical structure of the patient information is systematically mapped to the prompt parameters, with bracketed “[]" items from the (patient information) corresponding to (big category) classifications, while hyphenated “-" elements are allocated to (small category) designations. The referencing of the deep research prompt template incorporates outputs from deep research operations into the (deep research report) section, emulating the clinical reasoning process whereby practitioners consult and integrate contemporary research literature before formulating diagnostic conclusions. Sections marked as (omit) indicate portions where identical prompt text from previously described templates has been elided for clarity.**

**(Deep Research Prompt)**

You are a highly trained psychiatric research assistant.

Your goal is to investigate peer-reviewed journal articles about 12-week remission outcomes in depressive disorder patients receiving monotherapy. The following types of monotherapy medications may be considered: escitalopram, paroxetine, sertraline, duloxetine, venlafaxine, desvenlafaxine, milnacipran, mirtazapine, bupropion, vortioxetine, tianeptine, or trazodone.

You will focus on the following "(Big category)" variables and their possible relationship to remission rates:

(Small Category)

- Age, Sex ... (omitted) ... Homocysteine

1. Only consider peer-reviewed journal articles.
2. Exclude any articles by authors involved in the MAKE BETTER dataset.
3. Summarize your findings for each variable under the specified headings below.
4. If no evidence is found for a particular variable, write "None."
5. Provide a brief "Conclusion" section at the end, summarizing your overall findings.

Use the exact report format shown below, substituting only the bullet points with your findings or “None.” Then add the final “Conclusion” after these bullet points.

(Big Category)

- (Small Category)
- ...

Conclusion:

**(RoD prompt)**

You are an experienced ... (omitted) ... 2-week response, as well as a deep research report summarizing findings on 12-week remission outcomes for depressive disorder monotherapy.

Reason as needed, incorporating your own expertise and the research evidence contained in the deep research report below.

(Deep research report)

Your task:

1. ... (omitted) ...
2. ... (omitted) ...
3. ... (omitted) ...
4. Do not copy research text verbatim. Summarize relevant parts like a clinician referencing journal articles.
5. Final output format (follow precisely): ... (omitted) ...

The model was instructed to reference rather than directly replicate relevant insights from the “deep research report” when generating predictions and clinical rationales, thereby emulating the manner in which a practicing clinician would consult and synthesize findings from journal articles.

### Evaluation

For the 12-week remission prediction task, we designated “yes” as the positive class and “no” as the negative class. We computed balanced accuracy, sensitivity, specificity, positive predictive value (PPV), and negative predictive value (NPV) to compare quantitative performance. Additionally, to evaluate efficiency, we recorded both the inference generation cost and the average generation time (in seconds). The best-performing zero-shot model was selected based on balanced accuracy, reflecting the equal importance of both classes.

Additionally, we performed benchmarking analyses using logistic regression, random forest, and XGBoost models, evaluated through a patient-level stratified 15% hold-out design with repeated 10×5-fold cross-validation, reporting balanced accuracy, sensitivity, specificity, PPV, and NPV with 95% CIs across random seeds.

Subsequently, we applied the CoT, AoT, and RoD prompting methods to this best-performing model, compared their final performance using the same metrics, and further examined the medication-specific performance of the model that achieved the highest overall balanced accuracy.

Finally, 3 evaluators (2 psychiatry residents with >2 years of training and 1 psychiatrist specializing in depressive disorder with >10 y of experience) independently reviewed the clinical rationales and following treatment strategies generated for the correctly predicted cases by the best-performing model. They assessed these outputs across 5 domains (consistency, correctness, specificity, helpfulness, and human likeness) using a 5-point rating scale [34]. Consistency measured how closely the generated text aligned with the predicted answers, correctness evaluated its medical accuracy, specificity assessed its level of detail, helpfulness examined its clinical use, and human likeness considered how similar it was to typical human judgment.

## Results

### Baseline Demographics and Clinical Characteristics

Table 1 summarizes the baseline demographics and clinical characteristics across different monotherapy groups. The study population consisted of 244 patients prescribed SSRIs (escitalopram: n=159, 65%; paroxetine: n=60, 25%; and sertraline: n=25, 10%), 33 patients receiving SNRIs (duloxetine: n=20, 61%; venlafaxine: n=10, 30%; desvenlafaxine: n=2, 6%; and milnacipran: n=1, 3%), 99 patients on mirtazapine, 9 patients prescribed bupropion, and 5 patients taking other antidepressants (vortioxetine: n=3, 60%; tianeptine: n=1, 20%; and trazodone: n=1, 20%).

**Table 1.** Baseline demographics and clinical characteristics of preprocessed patients with depressive disorder, stratified according to the types of prescribed monotherapy (n=390).

Characteristics	Types of prescribed monotherapy				
	SSRI^a^ (n=244)	SNRI^b^ (n=33)	Mirtazapine (n=99)	Bupropion (n=9)	Others (n=5)
Sex, n (%)					
Female	175 (72)	25 (76)	77 (78)	4 (44)	4 (80)
Male	69 (28)	8 (24)	22 (22)	5 (56)	1 (20)
Employment status, n (%)					
Yes	180 (74)	25 (76)	67 (68)	7 (78)	4 (80)
No	64 (26)	8 (24)	32 (32)	2 (22)	1 (20)
Living alone, n (%)					
Yes	41 (17)	2 (6)	17 (17)	2 (22)	3 (60)
No	203 (83)	31 (94)	82 (83)	7 (78)	2 (40)
12-week remission, n (%)					
Yes	83 (34)	13 (39)	42 (42)	3 (33)	1 (20)
No	161 (66)	20 (61)	57 (58)	6 (67)	4 (80)
Age (y), mean (SD)	56.8 (14.5)	58.4 (9.5)	60.4 (14.1)	46.4 (14.6)	58.6 (8.0)
Height (cm), mean (SD)	159.9 (8.9)	157.3 (8.1)	159.1 (7.7)	165.1 (6.6)	157.9 (8.7)
Weight (kg), mean (SD)	59.7 (10.5)	58.3 (9.2)	59.4 (9.7)	59.0 (11.8)	60.7 (8.5)
HAM-D^c^, mean (SD)	20.4 (4.1)	20.5 (4.1)	21.2 (3.9)	18.6 (4.7)	22.2 (4.4)

a SSRI: selective serotonin reuptake inhibitor.

b SNRI: serotonin and norepinephrine reuptake inhibitor.

c HAM-D: the Hamilton Depression Rating Scale.

Among the total cohort (n=390), female participants constituted the majority (285/390, 73%), with similar gender distribution across the SSRIs (175/244, 72%), SNRIs (25/33, 76%), and mirtazapine groups (77/99, 78%). Employment was reported by 74% (180/244) of SSRI users, 76% (25/33) of SNRI users, and 68% (67/99) of mirtazapine users. At the 12-week assessment, 34% (83/244) of SSRI users, 39% (13/33) of SNRI users, and 42% (42/99) of mirtazapine users achieved remission. The mean baseline HAM-D scores ranged from 18.6 (SD 4.7) to 22.2 (4.4) points, with participants in the mirtazapine group being slightly older (mean 60.4, SD 14.1 y) than those in the bupropion group (mean 46.4, SD 14.6 y).

### Performance of Zero-Shot Prompting

The zero-shot performance section of Table 2 delineates the comparative outcomes of zero-shot experiments conducted with OpenAI’s ChatGPT o1 and o3-mini models across 3 distinct levels of reasoning effort, namely “low,” “medium,” and “high,” as well as for Anthropic’s Claude 3.7 Sonnet under 3 varying token budget settings (8000; 16,000; and 32,000 tokens). The findings indicate that all models demonstrated sensitivity values ranging from 0.6690 to 0.9085, suggesting that a significant proportion of patients who achieved remission were accurately identified. Conversely, specificity, which measures the correct identification of patients who did not achieve remission, exhibited lower values, ranging from 0.3185 to 0.6331 across the evaluated LLMs.

**Table 2.** Quantitative performance of zero-shot and advanced prompting techniques across 390 samples, including balanced accuracy, sensitivity, specificity, PPV,^a^ and NPV^b^.

Prompting, models, and reasoning parameters			Balanced accuracy	Sensitivity	Specificity	PPV	NPV	Time per generation (s)	Total cost (US $)
Zero-shot									
ChatGPT o1									
Low			0.6135	0.9085	0.3185	0.4329	0.8587	11.44	22.36
Medium			0.6382	0.9014	0.3750	0.4523	0.8692	19.63	35.20
High			0.6333	0.8592	0.4073	0.4535	0.8347	30.08	53.07
ChatGPT o3-mini									
Low			0.6121	0.8169	0.4073	0.4411	0.7953	4.84	1.14
Medium			0.6091	0.8028	0.4153	0.4402	0.7863	8.89	2.00
High			0.6323	0.8169	0.4476	0.4585	0.8102	20.43	4.39
Claude 3.7 Sonnet									
8000			0.6349	0.6972	0.5726	0.4829	0.7676	22.23	9.81
16,000			0.6511	0.6690	0.6331	0.5108	0.7696	23.78	10.90
32,000			0.6656	0.7183	0.6129	0.5152	0.7917	26.84	11.58
Zero-shot CoT^c^									
Claude 3.7 Sonnet with 32,000 tokens			0.6319	0.6549	0.6089	0.4895	0.7550	27.24	12.13
Zero-shot AoT^d^									
Claude 3.7 Sonnet with 32,000 tokens			0.6522	0.4859	0.8185	0.6053	0.7355	126.92	57.56
Zero-shot RoD^e^									
Claude 3.7 Sonnet with 32,000 tokens			0.6697	0.7183	0.6210	0.5204	0.7938	43.88	39.56

a PPV: positive predictive value.

b NPV: negative predictive value.

c CoT: chain-of-thoughts.

d AoT: atom-of-thoughts.

e RoD: referencing of deep research.

As the reasoning effort increased, all 3 models showed enhancements in both specificity and balanced accuracy. Specifically, the ChatGPT o1 model’s specificity improved from 0.3185 to 0.4073, with balanced accuracy rising from 0.6135 to 0.6333. Similarly, the ChatGPT o3-mini model experienced an increase in specificity from 0.4073 to 0.4476, alongside an improvement in balanced accuracy from 0.6121 to 0.6323. The Claude 3.7 Sonnet model also demonstrated an increase in specificity from 0.5726 to 0.6129, with a modest rise in balanced accuracy from 0.6349 to 0.6656.

From a computational efficiency standpoint, an increase in reasoning level generally resulted in heightened time and cost requirements across all models. Across all models evaluated, ChatGPT o1 incurred the highest overall costs, with total expenses ranging from $22.36 to $53.07. In contrast, ChatGPT o3-mini emerged as the most cost-effective option, with total costs between $1.14 and $4.39, rendering it the least expensive model. Furthermore, ChatGPT o3-mini exhibited superior speed efficiency, with task completion times ranging from 4.84 to 20.43 seconds, outperforming the other models in computational efficiency.

Conversely, Claude 3.7 Sonnet maintained a relatively stable computational profile across varying token budgets, with task completion times ranging from 22.23 seconds at the 8000-token setting to 26.84 seconds at the 32,000-token setting, and total costs increasing modestly from $9.81 to $11.58. Despite requiring more time per task than ChatGPT o3-mini at lower settings, Claude 3.7 Sonnet’s costs remained significantly lower than those of ChatGPT o1 at higher reasoning levels, while achieving the best overall performance, as evidenced by its balanced accuracy of 0.6656 at the 32,000-token reasoning budget. The detailed confusion matrices for all zero-shot prompting experiments are presented in Multimedia Appendix 3.

### Performance of Advanced Prompting

The advanced prompting (zero-shot CoT, AoT, and RoD) performance section of Table 2 outlines the performance metrics of 3 advanced prompt strategies applied to the Claude 3.7 Sonnet model using a 32,000-token reasoning budget, which demonstrated the best performance in the zero-shot context.

Among the advanced prompt strategies, the zero-shot CoT exhibited a balanced accuracy of 0.6319, with sensitivity and specificity values of 0.6549 and 0.6089, respectively, alongside a PPV of 0.4895 and an NPV of 0.7550. This performance is marginally lower than that of Claude 3.7 Sonnet’s zero-shot approach, particularly in terms of sensitivity and balanced accuracy.

The AoT strategy demonstrated a balanced accuracy of 0.6522, with a sensitivity of 0.4859 and a specificity of 0.8185. Its PPV and NPV were recorded at 0.6053 and 0.7355, respectively, while the time per task reached 126.92 seconds, and total costs escalated to $57.56, indicating a significant increase in computational resource demands compared to the zero-shot approach of Claude 3.7 Sonnet.

In contrast, the RoD approach achieved the highest balanced accuracy among the advanced prompts at 0.6697, with a sensitivity of 0.7183 and a specificity of 0.6210, slightly surpassing the performance of Claude 3.7 Sonnet’s zero-shot method. However, RoD’s time per task was approximately 1.63 times greater, and its total cost was approximately 3.42 times that of the zero-shot setting. The detailed confusion matrices for all advanced prompting experiments are presented in Multimedia Appendix 4.

For reference, conventional ML models trained on the numerically coded dataset achieved balanced accuracies ranging from 0.6077 to 0.7371 and sensitivities from 0.3533 to 0.6364 with overlapping 95% CIs (Multimedia Appendix 5).

### Medication-Specific Performance

Table 3 presents the performance metrics for the RoD strategy across various antidepressants, including SSRIs (escitalopram, paroxetine, and sertraline), SNRIs (duloxetine, venlafaxine, desvenlafaxine, and milnacipran), mirtazapine, bupropion, and others (vortioxetine, tianeptine, and trazodone), along with the number of correct predictions for both remission and nonremission outcomes. Among antidepressants with more than 50 cases, escitalopram (n=159), mirtazapine (n=99), and paroxetine (n=60) achieved balanced accuracies of 0.6799, 0.6873, and 0.6375, respectively.

**Table 3.** Quantitative performance of RoD^a^ prompting by medications, applied to Claude 3.7 Sonnet configured with 32,000 reasoning budget tokens.

Medications		Balanced accuracy	Sensitivity	Specificity	PPV^b^	NPV^c^	Correct predictions (yes), n/N	Correct predictions (no), n/N
SSRI^d^								
Escitalopram		0.6799	0.7407	0.6190	0.5000	0.8228	40/54	65/105
Paroxetine		0.6375	0.8000	0.4750	0.4324	0.8261	16/20	19/40
Sertraline		0.7083	0.6667	0.7500	0.6000	0.8000	6/9	12/16
SNRI^e^								
Duloxetine		0.6190	0.6667	0.5714	0.4000	0.8000	4/6	8/14
Venlafaxine		0.7083	0.7500	0.6667	0.6000	0.8000	3/4	4/6
Desvenlafaxine		0.5000	1.0000	0.0000	1.0000	0.0000	2/2	0/0
Milnacipran		0.0000	0.0000	0.0000	0.0000	0.0000	0/1	0/0
Mirtazapine		0.6873	0.6905	0.6842	0.6170	0.7500	29/42	39/57
Bupropion		0.7500	0.6667	0.8333	0.6667	0.8333	2/3	5/6
Others								
Vortioxetine		0.0000	0.0000	0.0000	0.0000	0.0000	0/1	0/2
Tianeptine		0.5000	0.0000	1.0000	0.0000	1.0000	0/0	1/1
Trazodone		0.5000	0.0000	1.0000	0.0000	1.0000	0/0	1/1

a RoD: referencing of deep research.

b PPV: positive predictive value.

c NPV: negative predictive value.

d SSRI: selective serotonin reuptake inhibitor.

e SNRI: serotonin and norepinephrine reuptake inhibitor.

### Medical Doctor Evaluation of Model-Generated Rationales and Treatment Strategies

A total of 3 clinical evaluators independently assessed the clinical rationales and treatment strategies generated by the best-performing model for 256 correctly predicted cases. As presented in Table 4, the highest total rating was observed for correctness (mean, 4.3, SD 0.7). Consistency, specificity, and helpfulness also received favorable evaluations (means 4.2, 4.2, and 4.2, respectively). Human likeness received the lowest but still positive rating (mean 3.6, SD 1.7). Notably, the board-certified psychiatrist rated helpfulness highest (mean 4.5, SD 0.6), while consistency scores varied most between evaluators, ranging from a mean of 3.4 to 4.9. To demonstrate the interpretability of the model’s reasoning process, one representative remission case (“yes”) and one nonremission case (“no”) were selected as examples, each accompanied by psychiatrist evaluations and comments. These illustrative cases are presented in MultimediaAppendices 6  7.

**Table 4.** Evaluations on clinical rationales and treatment strategies assigned by a board-certified psychiatrist and psychiatry residents for the clinical outputs produced by the best model across 256 correctly predicted cases.^a^

	Consistency, mean (SD)	Correctness, mean (SD)	Specificity, mean (SD)	Helpfulness, mean (SD)	Human likeness, mean (SD)
Psychiatrist	3.4 (0.6)	4.3 (0.5)	4.0 (0.5)	4.5 (0.6)	3.5 (0.5)
Resident 1	4.3 (0.5)	4.4 (0.7)	4.2 (0.6)	4.3 (0.7)	3.9 (2.6)
Resident 2	4.9 (0.4)	4.2 (0.8)	4.3 (0.8)	3.9 (1.3)	3.4 (1.2)
Residents^b^	4.6 (0.5)	4.3 (0.7)	4.3 (0.7)	4.1 (1.1)	3.6 (2.0)
Total^c^	4.2 (0.8)	4.3 (0.7)	4.2 (0.7)	4.2 (1.0)	3.6 (1.7)

a Assessments were conducted across 5 domains using a 5-point scale (1-5), with higher scores indicating better performance.

b The "residents" row represents the aggregated scores from both residents.

c "Total" indicates the combined assessment across all 3 evaluators.

## Discussion

### Principal Findings

Reasoning-based LLMs, especially when guided by research-informed prompting strategies, demonstrate promising potential in predicting antidepressant treatment response among patients with depressive disorder. To the best of our knowledge, this is among the first applications of LLMs for forecasting remission outcomes in depression, extending beyond prior approaches that primarily used traditional statistical and ML models [7-9,35,36].

In zero-shot contexts, all models showed higher sensitivity (0.6690‐0.9085) than specificity (0.3185‐0.6331). Balanced accuracy improved with enhanced reasoning: ChatGPT o1 by 3.22%, ChatGPT o3-mini by 3.3%, and Claude 3.7 Sonnet by 4.8%, with Claude achieving the highest performance (0.6656) at 32,000 budget tokens. This supports prior findings on reasoning capabilities’ importance in medical applications [37,38], suggesting that enhanced reasoning depth improves LLM performance in specific clinical tasks. Moreover, our proposed RoD technique, which emulates how clinicians incorporate contemporary research findings into their clinical reasoning process, outperformed zero-shot CoT and AoT with highest balanced accuracy (0.6697). While requiring further research, RoD appears effective for psychiatric prediction tasks. Compared with conventional ML baselines (Multimedia Appendix 5), which achieved balanced accuracies of 0.6077 to 0.7371 and sensitivities of 0.3533 to 0.6364, our reasoning-based LLM approach demonstrated higher sensitivity, indicating improved identification of patients who ultimately achieved remission. Analyzing medication-specific performance after excluding antidepressants with fewer than 10 cases, NPV remained high (>0.75) across all medications. For escitalopram, which was the most frequently prescribed antidepressant in the cohort (n=159), the RoD prompting approach achieved a balanced accuracy of 0.6799. Although direct comparison is limited by differences in sample size and methodology, this value is numerically higher than the 0.61 balanced accuracy reported in a prior partial least squares regression analysis of 92 escitalopram-treated patients [36], suggesting that reasoning-based LLMs may achieve comparable or potentially improved predictive capability within a single antidepressant group.

A particularly noteworthy finding is the contrasting performance between traditional reasoning approaches (CoT/AoT) and our knowledge-augmented RoD strategy. While CoT and AoT showed minimal improvement or even slight performance degradation compared to zero-shot prompting, RoD achieved consistent improvements across all metrics. This divergence suggests that for clinical pattern-recognition tasks, the decomposition of reasoning steps alone (as in CoT/AoT) may introduce unnecessary complexity without meaningful benefit. In contrast, RoD’s incorporation of synthesized research evidence appears to provide crucial contextual priors that enhance prediction accuracy. This mirrors actual clinical practice, where psychiatrists integrate empirical evidence from literature with patient-specific data rather than relying solely on sequential logical reasoning.

The superior performance of RoD likely stems from its ability to leverage documented patterns in depressive disorder treatment outcomes, effectively providing the model with a knowledge base of established clinical associations. This approach compensates for the inherent limitations of LLMs in medical domains, where training data may not adequately capture the full spectrum of clinical scenarios. Furthermore, by grounding predictions in research evidence, RoD may reduce the risk of hallucinations or clinically implausible outputs that can occur with pure reasoning approaches, a critical concern in medical artificial intelligence (AI) applications [37]. These findings align with recent evidence suggesting that retrieval-augmented approaches enhance LLM reliability in clinical contexts [38]. The hybrid strategy combining LLM reasoning with structured knowledge integration may represent an optimal approach for clinical prediction tasks, particularly in psychiatry, where outcomes are influenced by complex biopsychosocial factors [39].

### Clinical Implications

Clinical evaluation of the model-generated rationales and treatment suggestions revealed high ratings for correctness, consistency, specificity, and perceived helpfulness, indicating that reasoning-based LLMs can produce clinically coherent and contextually relevant outputs. Favorable assessments by practicing clinicians further suggest their potential as valuable adjuncts in real-world clinical decision-making, particularly for the early identification of patients at risk of treatment nonremission. Unlike prior models focused mainly on predictive performance, our approach emphasizes interpretability and clinician usability, which are key elements for real-world application. By integrating biomarker and clinical data with advanced reasoning, LLMs may support more personalized and effective treatment decisions. Nonetheless, relatively lower ratings for human likeness highlight the need for improved communication style to foster trust and interpretability in clinical practice.

The high NPV (>0.75) across all medication classes suggests particular utility as a screening tool to identify patients unlikely to achieve remission with standard first-line treatments. This could enable a stratified care approach, where predicted nonresponders receive enhanced monitoring, earlier treatment adjustments, or augmentation strategies, potentially reducing the typical 12-week trial-and-error period. Such implementation aligns with recent frameworks for integrating AI into clinical psychiatry that emphasize augmentation rather than replacement of clinical judgment [40]. The RoD prompting strategy required an average processing time of 43.88 seconds per patient, suggesting that real-time clinical application is feasible within standard consultation time frames.

From a health economics perspective, early identification of nonresponders could substantially reduce costs associated with prolonged ineffective treatments, emergency interventions, and productivity losses. The ability to provide detailed clinical rationales distinguishes our approach from black-box algorithms, addressing a critical barrier to AI adoption in psychiatry, where understanding the reasoning behind recommendations is essential for clinical acceptance and regulatory approval [41]. Moreover, the cloud-based nature of LLMs enables deployment without specialized hardware, making this technology accessible to resource-limited settings where psychiatric expertise may be scarce [42].

Successful clinical implementation would require integration with electronic health records, development of user-friendly interfaces, and establishment of clear protocols for acting on model predictions. The model’s ability to suggest alternative treatment strategies when predicting nonremission provides actionable guidance rather than mere risk stratification, potentially improving clinical utility. Furthermore, the transparent reasoning process could serve an educational function, helping less experienced clinicians understand factors influencing treatment response and potentially improving their clinical reasoning skills over time [43]. Prospective validation studies are warranted to confirm these findings in real-world clinical settings.

### Limitations

Despite promising findings, several limitations warrant consideration. First, while our approach demonstrated robust sensitivity (0.7183) and NPV (0.7938), the relatively low PPV (0.5204) may generate false positives, potentially complicating treatment planning for patients misclassified as achieving remission [44]. The relatively modest PPV observed in our model should be interpreted in light of the low remission prevalence in our cohort, a condition known to constrain PPV despite adequate discriminative performance. Although PPV was modest, the model demonstrated balanced accuracy and sensitivity at clinically meaningful levels, supporting its capacity for reliable risk stratification in a heterogeneous depressive population. Importantly, the high NPV suggests that the model may be particularly effective for identifying patients unlikely to achieve remission, thereby enabling early treatment modifications or augmentation strategies to improve outcomes. These findings emphasize that the model is intended as an adjunctive decision-support tool, and its predictions should be integrated with comprehensive clinical assessments.

Medication-specific analyses revealed sample imbalances (Table 3), with escitalopram dominating (n=159) and several medications having fewer than 20 cases. Although overall model performance remained robust, medication-specific metrics should be interpreted with caution for drugs with limited samples. This imbalance reflects real-world prescribing patterns but limits our ability to make definitive conclusions about model performance for less commonly prescribed antidepressants [45]. Future studies should either focus on medications with adequate sample sizes or use targeted recruitment strategies to ensure sufficient representation across all medication classes.

Our clinical evaluation methodology has notable limitations. The assessment was conducted by only 3 evaluators from a single institution, potentially introducing institutional bias and limiting generalizability. More critically, evaluation was restricted to correctly predicted cases, which likely inflates perceived quality scores and fails to capture model behavior in misclassification scenarios. Future studies should incorporate multi-institutional evaluators and a comprehensive assessment of both correct and incorrect predictions to provide more robust validation of AI-assisted diagnostic approaches.

Finally, the RoD method requires further comparative evaluation against alternative knowledge-augmented techniques to determine its optimal application in psychiatric contexts. Validation in ethnically diverse populations with larger numbers of clinical expert appraisals remains essential. Prospective randomized trials are needed to evaluate whether model recommendations improve clinical outcomes and decision-making in practice.

### Conclusions

In conclusion, this study demonstrates the promising potential of reasoning-based LLMs for predicting antidepressant treatment response in patients with depressive disorder. Our findings highlight the superior performance of the RoD technique, which achieved the highest performance by integrating research evidence with clinical reasoning, representing an important advance toward AI-assisted clinical decision support in psychiatry. The high NPV (>0.75) across medications suggests particular use as a screening tool for identifying patients unlikely to achieve remission with standard treatments. While limitations exist, including the need for validation in diverse populations and larger-scale clinical evaluations, the positive assessment by clinical experts validates the potential use of these approaches. Future research should focus on expanding real-world treatment outcome datasets, conducting multi-institutional clinical evaluations, and developing models that can predict both the magnitude of treatment response and suggest personalized next-step strategies. These advances could enable clinicians to make more informed, evidence-based decisions in selecting the most effective personalized treatment strategies for patients with depressive disorder.

## Supplementary material

10.2196/83352 Multimedia Appendix 1 Supplementary materials on the MAKE BETTER study.

10.2196/83352 Multimedia Appendix 2 Structure of the atom-of-thoughts (AoT) prompt.

10.2196/83352 Multimedia Appendix 3 Confusion matrices for each zero-shot prompting under varying reasoning levels or token budgets.

10.2196/83352 Multimedia Appendix 4 Confusion matrices for advanced prompting strategies.

10.2196/83352 Multimedia Appendix 5 Predictive performance of machine learning models for 12-week remission classification.

10.2196/83352 Multimedia Appendix 6 Representative remission (“yes”) case generated by the RoD prompting strategy.

10.2196/83352 Multimedia Appendix 7 Representative remission (“no”) case generated by the RoD prompting strategy.

## Abbreviations

AI: artificial intelligence
AoT: atom-of-thoughts
CoT: chain-of-thoughts
HAM-D: Hamilton Depression Rating Scale
LLM: large language model
MAKE BETTER study: MAKE Biomarker Discovery for Enhancing Antidepressant Treatment Effect and Response study
ML: machine learning
NPV: negative predictive value
PPV: positive predictive value
RoD: referencing of deep research
SNRI: serotonin and norepinephrine reuptake inhibitor
SSRI: selective serotonin reuptake inhibitors

## References

[1] GBD 2019 Mental Disorders Collaborators (2022). Global, regional, and national burden of 12 mental disorders in 204 countries and territories, 1990–2019: a systematic analysis for the Global Burden of Disease Study 2019. Lancet Psychiatry.

[2] Kim JM, Kim SW, Stewart R (2011). Predictors of 12-week remission in a nationwide cohort of people with depressive disorders: the CRESCEND study. Hum Psychopharmacol.

[3] Jin YT, Kim HY, Jhon M (2022). Prediction of 12-Week remission by psychopharmacological treatment step in patients with depressive disorders. Psychiatry Investig.

[4] Walter HJ, Abright AR, Bukstein OG (2023). Clinical practice guideline for the assessment and treatment of children and adolescents with major and persistent depressive disorders. J Am Acad Child Adolesc Psychiatry.

[5] Perlman K, Benrimoh D, Israel S (2019). A systematic meta-review of predictors of antidepressant treatment outcome in major depressive disorder. J Affect Disord.

[6] Sharma A, Barrett MS, Cucchiara AJ, Gooneratne NS, Thase ME (2017). A breathing-based meditation intervention for patients with major depressive disorder following inadequate response to antidepressants: a randomized pilot study. J Clin Psychiatry.

[7] Benoit JRA, Dursun SM, Greiner R (2022). Using machine learning to predict remission in patients with major depressive disorder treated with desvenlafaxine. Can J Psychiatry.

[8] Salem H, Huynh T, Topolski N (2023). Temporal multi-step predictive modeling of remission in major depressive disorder using early stage treatment data; STAR*D based machine learning approach. J Affect Disord.

[9] Carr E, Rietschel M, Mors O (2025). Optimizing the prediction of depression remission: a longitudinal machine learning approach. Am J Med Genet B Neuropsychiatr Genet.

[10] Zhukovsky P, Trivedi MH, Weissman M, Parsey R, Kennedy S, Pizzagalli DA (2025). Generalizability of treatment outcome prediction across antidepressant treatment trials in depression. JAMA Netw Open.

[11] Cheng SW, Chang CW, Chang WJ (2023). The now and future of ChatGPT and GPT in psychiatry. Psychiatry Clin Neurosci.

[12] Shin D, Kim H, Lee S, Cho Y, Jung W (2024). Using large language models to detect depression from user-generated diary text data as a novel approach in digital mental health screening: instrument validation study. J Med Internet Res.

[13] Omar M, Soffer S, Charney AW, Landi I, Nadkarni GN, Klang E (2024). Applications of large language models in psychiatry: a systematic review. Front Psychiatry.

[14] (2024). Introducing openai o1-preview. OpenAI.

[15] (2025). OpenAI o3-mini: pushing the frontier of cost-effective reasoning. OpenAI.

[16] (2025). Claude's extended thinking. Anthropic.

[17] Xie Y, Wu J, Tu H, Yang S, Zhao B, Zong Y (2024). A preliminary study of o1 in medicine: are we closer to an ai doctor?. arXiv.

[18] Chen J, Cai Z, Ji K, Wang X, Liu W, Wang R (2024). Huatuogpt-o1, towards medical complex reasoning with llms. arXiv.

[19] Mondillo G, Colosimo S, Perrotta A, Frattolillo V, Masino M (2025). Comparative evaluation of advanced AI reasoning models in pediatric clinical decision support: chatgpt O1 vs. deepseek-r1. medRxiv.

[20] Mondillo G, Masino M, Colosimo S, Perrotta A, Frattolillo V (2025). Evaluating AI reasoning models in pediatric medicine: a comparative analysis of o3-mini and o3-mini-high. medRxiv.

[21] Xu P, Wu Y, Jin K, Chen X, He M, Shi D (2025). DeepSeek-R1 outperforms Gemini 2.0 Pro, OpenAI o1, and o3-mini in bilingual complex ophthalmology reasoning. Adv Ophthalmol Pract Res.

[22] Kang HJ, Kim JW, Kim SY (2018). The MAKE biomarker discovery for enhancing antidepressant treatment effect and response (MAKE BETTER) study: design and methodology. Psychiatry Investig.

[23] Sheehan DV, Lecrubier Y, Sheehan KH (1998). The MINI-International Neuropsychiatric Interview (M.I.N.I.): the development and validation of a structured diagnostic psychiatric interview for DSM-IV and ICD-10. J Clin Psychiatry.

[24] Overall JE, Gorham DR (1962). The brief psychiatric rating scale. Psychol Rep.

[25] HAMILTON M (1960). A rating scale for depression. J Neurol Neurosurg Psychiatry.

[26] Rabin R, de Charro F (2001). EQ-5D: a measure of health status from the EuroQol Group. Ann Med.

[27] Sheehan DV (1983). The Anxiety Disease.

[28] Cohen S, Kamarck T, Mermelstein R (1983). A global measure of perceived stress. J Health Soc Behav.

[29] Connor KM, Davidson JRT (2003). Development of a new resilience scale: the Connor-Davidson Resilience Scale (CD-RISC). Depress Anxiety.

[30] Zimet GD, Dahlem NW, Zimet SG, Farley GK (1988). The multidimensional scale of perceived social support. J Pers Assess.

[31] Kojima T, Gu SS, Reid M, Matsuo Y, Iwasawa Y (2022). Large language models are zero-shot reasoners. https://dl.acm.org/doi/10.5555/3600270.3601883.

[32] Teng F, Yu Z, Shi Q, Zhang J, Wu C, Luo Y (2025). Atom of thoughts for markov llm test-time scaling. arXiv.

[33] (2025). Introducing deep research. OpenAI.

[34] Kwon T, Ong KT, Kang D (2024). Large language models are clinical reasoners: reasoning-aware diagnosis framework with prompt-generated rationales. https://ojs.aaai.org/index.php/AAAI/article/view/29802.

[35] Wallert J, Boberg J, Kaldo V (2022). Predicting remission after internet-delivered psychotherapy in patients with depression using machine learning and multi-modal data. Transl Psychiatry.

[36] LoParo D, Dunlop BW, Nemeroff CB, Mayberg HS, Craighead WE (2025). Prediction of individual patient outcomes to psychotherapy vs medication for major depression. Npj Ment Health Res.

[37] Omiye JA, Lester JC, Spichak S, Rotemberg V, Daneshjou R (2023). Large language models propagate race-based medicine. NPJ Digit Med.

[38] Zakka C, Shad R, Chaurasia A (2024). Almanac - retrieval-augmented language models for clinical medicine. NEJM AI.

[39] Borrell-Carrió F, Suchman AL, Epstein RM (2004). The biopsychosocial model 25 years later: principles, practice, and scientific inquiry. Ann Fam Med.

[40] Topol EJ (2019). High-performance medicine: the convergence of human and artificial intelligence. Nat Med.

[41] Wiens J, Saria S, Sendak M (2019). Do no harm: a roadmap for responsible machine learning for health care. Nat Med.

[42] Thirunavukarasu AJ, Ting DSJ, Elangovan K, Gutierrez L, Tan TF, Ting DSW (2023). Large language models in medicine. Nat Med.

[43] Beam AL, Manrai AK, Ghassemi M (2020). Challenges to the reproducibility of machine learning models in health care. JAMA.

[44] Obradovich N, Khalsa SS, Khan W (2024). Opportunities and risks of large language models in psychiatry. NPP Digit Psychiatry Neurosci.

[45] Bzdok D, Meyer-Lindenberg A (2018). Machine learning for precision psychiatry: opportunities and challenges. Biol Psychiatry Cogn Neurosci Neuroimaging.

