# Supplementary material for: Prediction of 12-Week Remission in Patients With Depressive Disorder Using Reasoning-Based Large Language Models: Model Development and Validation Study
Source: JMIR Ment Health. 2026 Jan 23;13:e83352. doi: 10.2196/83352 (PMC12829737; doi:10.2196/83352)
Supplement: Multimedia Appendix 1 [file mental-v13-e83352-s001.docx]

**Multimedia Appendix 1. Supplementary materials on the MAKE BETTER study**

| **Study outline** |
| --- |
| This study was defined *a priori* and comprised the primary component of the MAKE Biomarker discovery for Enhancing anTidepressant Treatment Effect and Response (MAKE BETTER) project, which intends to develop a treatment-response prediction index composed of biomarkers for patients with depressive disorders. Study details have been published as a protocol paper [1] and registered with cris.nih.go.kr (identifier: KCT0001332). To reflect real-world settings, participants enrolment and treatment interventions were conducted in a naturalistic fashion. This study was approved by the Chonnam National University Hospital Institutional Review Board (CNUH 2012-014). Reflecting true clinical situations, recruitment was inclusive of various depression subtypes and comorbid physical conditions. Treatment was administered in a naturalistic manner, allowing flexibility in medication type, dosage, and regimen based on patient and clinician preferences, structured around predefined intervals and measurement points. During the initial 3-week phase of antidepressant monotherapy, subsequent adjustments were permitted at 3-week intervals up to 12 weeks after treatment initiation. Data collection on biomarkers and other variables was conducted via a structured clinical report form by coordinators unaware of the treatment details at baseline, following training by psychiatric researchers.  This study was approved by the Chonnam National University Hospital Institutional Review Board (CNUH 2012-014). Written informed consent was obtained from all participants. For minors, parental permission and child assent would have been required under institutional and national regulations; however, no minors were enrolled in this study.  All participant data were de-identified and assigned unique study codes. Personal identifiers were stored separately from clinical data in secure, password-protected databases accessible only to authorized research personnel. Participants received no financial compensation for their participation in this study. |
| **Eligibility criteria** |
| Inclusion criteria were: i) aged ≥7 years (to reflect real-world depressive outpatients across all age ranges); ii) diagnosed with MDD, dysthymic disorder, or depressive disorder not otherwise specified (NOS), using the Mini-International Neuropsychiatric Interview (MINI) [2], a diagnostic psychiatric interview applying Diagnostic and Statistical Manual of Mental Disorders, Fourth Edition (DSM-IV) [3] criteria; iii) Hamilton Depression Rating Scale (HAMD) [4] (score ≥ 14; iv) able to complete questionnaires, understand the objective of the study, and sign the informed consent form. However, all participants who were actually enrolled and analyzed in this study were adults (≥17 years); no minors were included due to the requirements for parental permission, child assent, and safety considerations for antidepressant use in minors. Exclusion criteria were: i) an unstable or uncontrolled medical condition; ii) unable to complete the psychiatric assessment or comply with the medication regimen, due to a severe physical illness; iii) current or lifetime DSM-IV diagnosis of bipolar disorder, schizophrenia, schizoaffective disorder, schizophreniform disorder, psychotic disorder NOS, or other psychotic disorder; iv) history of organic psychosis, dementia, epilepsy, or seizure disorder; v) history of anticonvulsant treatment; vi) hospitalization for any psychiatric diagnosis apart from depressive disorder (e.g., alcohol/drug dependence); vii) electroconvulsive therapy received for the current depressive episode; viii) pregnant or breastfeeding. |
| **Stepwise Pharmacotherapy** |
| Before treatment commencement, a comprehensive review was made of the patients’ clinical manifestations (e.g., psychotic and anxiety symptoms), severity of illness, physical comorbidities and medication profiles, and history of previous treatments. Minimal and maximal dosages of pharmacological agents were determined considering existing treatment guidelines [5, 6] In the first treatment Step 1, patients received antidepressant treatment, taking into consideration these data and treatment guidelines [6-8], for 3 weeks. Antidepressants used were bupropion, desvenlafaxine, duloxetine, escitalopram, fluoxetine, mirtazapine, paroxetine, sertraline, venlafaxine, and vortioxetine. After Step 1 antidepressant monotherapy, next step pharmacotherapy could be administered every 3 weeks during the acute treatment phase (3, 6, 9, and 12 weeks with a 3-day allowable window), whenever needed. At the end of each step, overall effectiveness and tolerability were reviewed for proceeding with measurement-based next-step treatments. In cases of insufficient improvement (a HAMD score reduction of <30% from the baseline) or intolerable side effects, patients were instructed to choose whether they would prefer to remain in the current step or enter the next step strategies with switching (S), augmentation (A), combination (C), S + A, S + C, A + C, and S + A + C treatment. Patients were also allowed to receive next step treatment if they showed sufficient improvement (a HAMD score reduction of ≥ 30% from the baseline) and absent/tolerable side effects. For determining treatment strategies, each patient’s preference was given priority to maximize medication compliance and treatment outcomes [9]. Antidepressants switched or combined were bupropion, desvenlafaxine, duloxetine, escitalopram, fluoxetine, mirtazapine, paroxetine, sertraline, venlafaxine, and vortioxetine. Augmented drugs were buspirone, lithium, triiodothyronine, and atypical antipsychotics including aripiprazole, risperidone, olanzapine, quetiapine, and ziprasidone. Although the stepwise pharmacotherapy was conducted in MAKEBETTER study, the present analyses included depressive patients who maintained first-step monotherapy for 12 weeks. |
| **Variables** |
| Socio-demographic data included age, sex, cohabitation status (living alone or not), occupational status (current employed or not). Clinical characteristics encompassed response to the 9-item Mini-International Neuropsychiatric Interview (MINI) [2] depression specifiers based on DSM-IV criteria (melancholic, atypical features and psychotic features) [3], age at onset, illness duration, number of previous depressive episodes, duration of current episode. Additional information included family history of depression, number of concurrent physical disorders (based on a checklist of 15 conditions or systems), and smoking status (current smoker or not).  Baseline depressive symptoms were assessed using the Hamilton Depression Rating Scale (HAMD) [4], anxiety using the Hospital Anxiety Depression Scale-Anxiety subscales (HADS-A) [10], and alcohol related problems via the Alcohol Use Disorders Identification Test (AUDIT) [11]. Health-related quality of life was measured using the EuroQol-5 Dimension (EQ-5D) [12], functional impairment with the Sheehan Disability Scale (SDS) [13], perceived stress with Perceived Stress Scale (PSS) [14], resilience with the Conner-Davidson Resilience Scale (CD-RISC) [15], and perceived social support with the Multidimensional Scale of Perceived Social Support (MSPSS) [16].  Higher scores indicated greater symptom severity except for EQ-5D, CD-RISC and MSPSS where higher scores indicated better quality of life, resilience, and social support. For female participants, fertility- and depression-related variables included age at menarche or menopause, hormonal therapy use, and presence of peri-/postpartum or postmenopausal depression.  Early response (ER), defined as ≥20% reduction in HAM-D scores at two weeks, was included as variables given its known relevance to 12-weeks remission outcome [17]. |
| **Biomarker Assessments** |
| Participants were instructed to fast from the night before for morning blood sampling, and to sit for 25-45 min quietly and relax before blood samples were acquired. Blood (10ml) was collected into dry tubes and stored immediately in a refrigerator between 2 and 4°C from 3 to 6 hours. Centrifugation (3000×g for 15 min at 4 °C) was performed at the day of blood sampling, and serum samples were then immediately frozen at −80°C at clinical laboratories of the CNUH.  All laboratory measurements were conducted by the Global Clinical Central Lab (Yongin, Korea) blind to patients’ status. Samples were unfrozen just before the measurements, which were run at the same time to avoid multiple freeze/thaw cycles and batch effects, respectively. Fourteen blood biomarkers representing six functional systems were selected based upon our literature search and meta-review [18].  Blood biomarkers were measured using the following methods:  i) HPA axis   - cortisol: Cobas Cortisol II electrochemiluminescence Immunoassay (Roche, Vilvoorde, Belgium).   ii) Immune   - hsCRP: Tina-quant C-reactive protein (latex) high sensitive assay (Roche, Vilvoorde, Belgium). - TNF-α: Quantikine^®^ HS ELISA Human TNF-α Immunoassay (R&D Systems, Minneapolis, USA). - IL-1β, IL-6, IL-4, and IL-10: Human High Sensitivity T Cell Magnetic Bead Panel (EMD Millipore, Billerica, USA).   iii) Metabolic   - leptin: Human Leptin ELISA (BioVendor Laboratory Medicine, Inc., Modrice, Czech Republic). - total ghrelin: GHRELIN (Total) radioimmunoassay kit (EMD Millipore, Billerica,USA). - total cholesterol: L-type CHO M cholesterol oxidase method kit (Wako Pure Chemical Industries, Osaka, Japan).   iv) Neurotransmitter   - serotonin: ClinRep high-performance liquid chromatography kit (Recipe, Munich, Germany).   v) Neurogenic or neuroplastic   - BDNF: Quantikine^®^ ELISA Human BDNF Immunoassay (R&D Systems Inc., Minneapolis, USA).   vi) Nutritional   - folate: Cobas Elecsys Folate III electrochemiluminescence Immunoassay (Roche, Vilvoorde, Belgium).   homocysteine: ARCHITECT Homocysteine 1L71 Kit (Abbot, Wiesbaden, Germany). |

**Abbreviations**: MAKE BETTER study, the MAKE Biomarker Discovery for Enhancing Antidepressant Treatment Effect and Response study.

***Note***:

1. Kang H-J, Kim J-W, Kim S-Y, Kim S-W, Shin H-Y, Shin M-G, et al. The MAKE Biomarker discovery for Enhancing anTidepressant Treatment Effect and Response (MAKE BETTER) study: design and methodology. Psychiatry Investigation. 2018;15(5):538.

2. Sheehan DV, Lecrubier Y, Sheehan KH, Amorim P, Janavs J, Weiller E, et al. The Mini-International Neuropsychiatric Interview (MINI): the development and validation of a structured diagnostic psychiatric interview for DSM-IV and ICD-10. J clin psychiatry. 1998;59(Suppl 20):22-33.

3. Diagnostic A. Statistical Manual of Mental disorders, 4th edition American Psychiatric Association. Washington, DC. 1994.

4. Hamilton M. A rating scale for depression. Journal of neurology, neurosurgery, and psychiatry. 1960;23(1):56.

5. Anderson I, Ferrier I, Baldwin R, Cowen P, Howard L, Lewis G, et al. Evidence-based guidelines for treating depressive disorders with antidepressants: a revision of the 2000 British Association for Psychopharmacology guidelines. Journal of psychopharmacology. 2008;22(4):343-96.

6. Bauer M, Pfennig A, Severus E, Whybrow PC, Angst J, Möller H-J, et al. World Federation of Societies of Biological Psychiatry (WFSBP) guidelines for biological treatment of unipolar depressive disorders, part 1: update 2013 on the acute and continuation treatment of unipolar depressive disorders. The world journal of biological psychiatry. 2013;14(5):334-85.

7. Kennedy SH, Lam RW, McIntyre RS, Tourjman SV, Bhat V, Blier P, et al. Canadian Network for Mood and Anxiety Treatments (CANMAT) 2016 clinical guidelines for the management of adults with major depressive disorder: section 3. Pharmacological treatments. The Canadian Journal of Psychiatry. 2016;61(9):540-60.

8. Malhi GS, Bassett D, Boyce P, Bryant R, Fitzgerald PB, Fritz K, et al. Royal Australian and New Zealand College of Psychiatrists clinical practice guidelines for mood disorders. Australian & New Zealand Journal of Psychiatry. 2015;49(12):1087-206.

9. Swift JK, Callahan JL. The impact of client treatment preferences on outcome: A meta‐analysis. Journal of clinical psychology. 2009;65(4):368-81.

10. Zigmond AS, Snaith RP. The hospital anxiety and depression scale. Acta psychiatrica scandinavica. 1983;67(6):361-70.

11. Saunders JB, Aasland OG, Babor TF, De la Fuente JR, Grant M. Development of the alcohol use disorders identification test (AUDIT): WHO collaborative project on early detection of persons with harmful alcohol consumption‐II. Addiction. 1993;88(6):791-804.

12. Rabin R, Charro Fd. EQ-5D: a measure of health status from the EuroQol Group. Annals of medicine. 2001;33(5):337-43.

13. Sheehan D. The anxiety disease. 1983. New York, NY: Charles Scribner's Sons. 1986:144-53.

14. Cohen S, Kamarck T, Mermelstein R. A global measure of perceived stress. Journal of health and social behavior. 1983:385-96.

15. Connor KM, Davidson JR. Development of a new resilience scale: The Connor‐Davidson resilience scale (CD‐RISC). Depression and anxiety. 2003;18(2):76-82.

16. Zimet GD, Dahlem NW, Zimet SG, Farley GK. The multidimensional scale of perceived social support. Journal of personality assessment. 1988;52(1):30-41.

17. Szegedi A, Jansen WT, van Willigenburg AP, van der Meulen E, Stassen HH, Thase ME. Early improvement in the first 2 weeks as a predictor of treatment outcome in patients with major depressive disorder: a meta-analysis including 6562 patients. Journal of Clinical Psychiatry. 2009;70(3):344.

18. Kennis M, Gerritsen L, van Dalen M, Williams A, Cuijpers P, Bockting C. Prospective biomarkers of major depressive disorder: a systematic review and meta-analysis. Molecular psychiatry. 2020;25(2):321-38.
