# Supplementary material for: Prediction of 12-Week Remission in Patients With Depressive Disorder Using Reasoning-Based Large Language Models: Model Development and Validation Study
Source: JMIR Ment Health. 2026 Jan 23;13:e83352. doi: 10.2196/83352 (PMC12829737; doi:10.2196/83352)
Supplement: Multimedia Appendix 2 [file mental-v13-e83352-s002.docx]

**Multimedia Appendix 2. Structure of the atom of thoughts prompt**


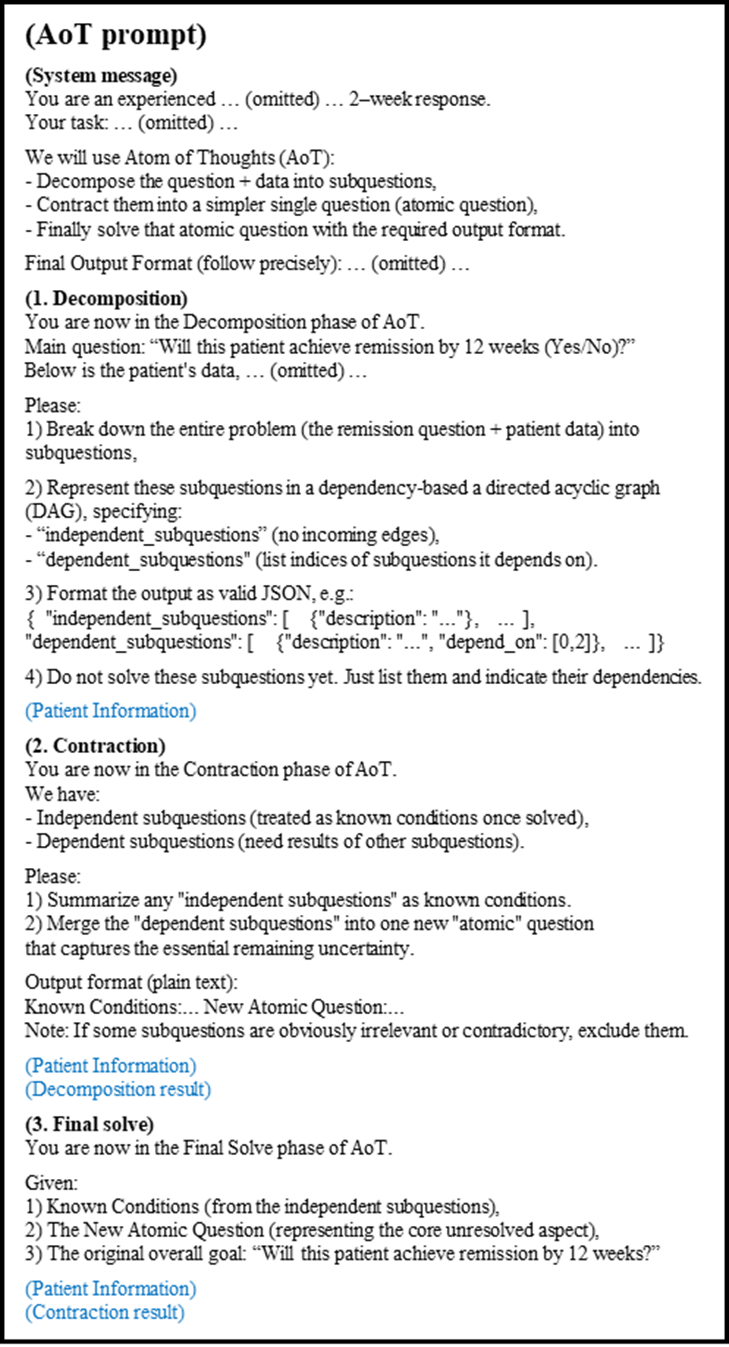


**Abbreviations**: AoT, atom of thoughts.

***Note***: This figure presents the prompt structure for the AoT technique implemented in this study. Sections marked with {omit} contain identical text from the zero-shot prompt. We adapted the conventional AoT methodology to align with our specific task requirements. Throughout experimentation, the prompt template remained consistent while only the {Patient Information} section was systematically substituted with individual patient data. The corresponding {Decomposition result} and {Contraction result} fields were populated with model-generated outputs tailored to each patient's clinical profile.
