# Supplementary material for: Prediction of 12-Week Remission in Patients With Depressive Disorder Using Reasoning-Based Large Language Models: Model Development and Validation Study
Source: JMIR Ment Health. 2026 Jan 23;13:e83352. doi: 10.2196/83352 (PMC12829737; doi:10.2196/83352)
Supplement: Multimedia Appendix 3 [file mental-v13-e83352-s003.docx]

Multimedia Appendix 3. Confusion matrices for each zero-shot prompting under varying reasoning levels or token budgets


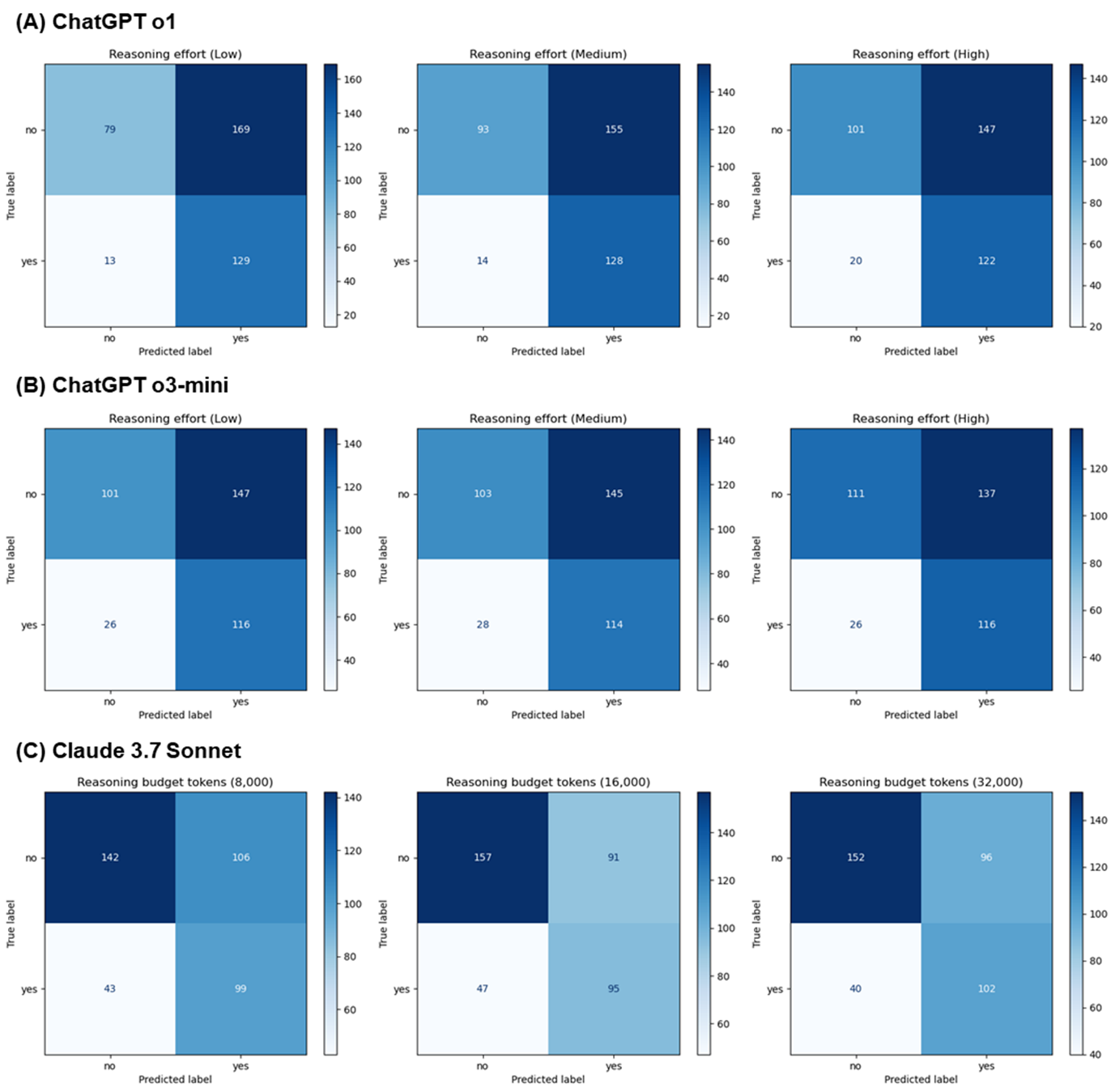


***Note***: This figure presents the confusion matrices for each model by zero-shot prompting, offering a comprehensive view of their performance in the remission prediction task. For ChatGPT o1, an increase in reasoning effort from "low" to "high" resulted in an increase in true negatives (TN) from 79 to 101, while true positives (TP) slightly decreased from 129 to 122. In the case of ChatGPT o3-mini, elevating the reasoning effort led to an increase in TN from 101 to 111, with TP remaining constant at 116. In contrast, Claude 3.7 Sonnet demonstrated a higher prediction accuracy for the negative class, with TN values ranging from 142 to 152, although its TP values were slightly lower, fluctuating between 99 and 102. Ultimately, Claude 3.7 Sonnet, with a 32,000-token reasoning budget, achieved the highest total of correct predictions (TN + TP = 254), indicating superior performance in accurately identifying both patients who did not achieve remission and those who did.
