# Supplementary material for: Prediction of 12-Week Remission in Patients With Depressive Disorder Using Reasoning-Based Large Language Models: Model Development and Validation Study
Source: JMIR Ment Health. 2026 Jan 23;13:e83352. doi: 10.2196/83352 (PMC12829737; doi:10.2196/83352)
Supplement: Multimedia Appendix 4 [file mental-v13-e83352-s004.docx]

Multimedia Appendix 4. Confusion Matrices for Advanced Prompting Strategies


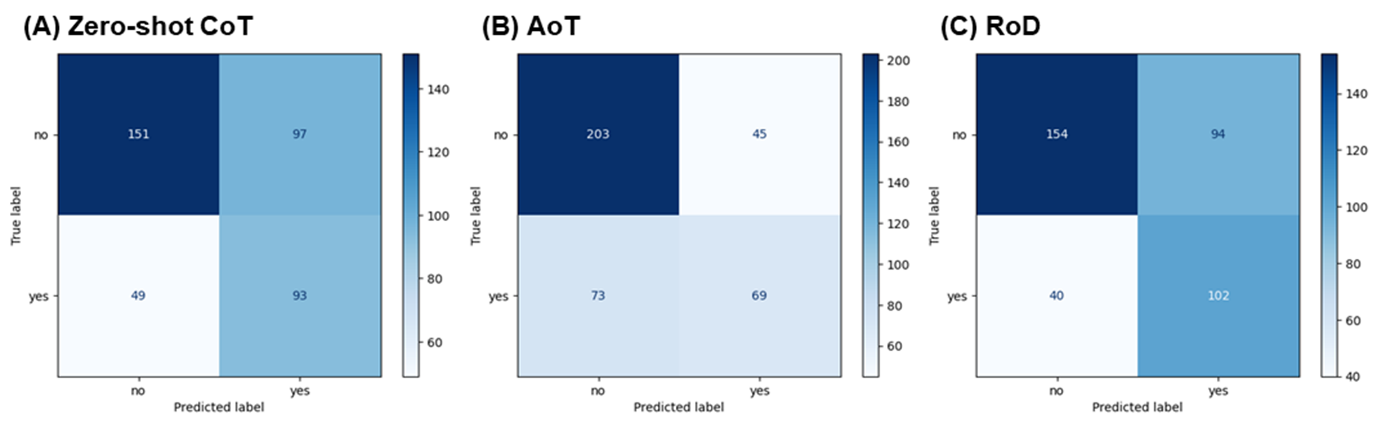


**Abbreviations**: CoT, chain of thoughts; AoT, atom of thoughts; RoD, referencing of deep research.

***Note***: This figure illustrates the confusion matrices for these advanced prompt strategies, providing a detailed view of their performance in the remission prediction task. For Zero-shot CoT, the confusion matrix revealed 151 true negatives (TN), 93 true positives (TP), 49 false negatives (FN), and 97 false positives (FP), indicating a slightly lower performance compared to the zero-shot approach. The AoT model achieved the highest prediction accuracy for the non-remission group, with 203 TN, but exhibited the poorest prediction performance for the remission group, with only 69 TP. For RoD, the confusion matrix indicated 154 TN and 102 TP, suggesting that while its performance did not significantly exceed that of the zero-shot approach, it avoided the performance decline observed in other advanced prompts. Moreover, RoD achieved the highest total of correct predictions (TN + TP = 256), demonstrating the highest accuracy rate among the evaluated strategies.
