# Supplementary material for: Prediction of 12-Week Remission in Patients With Depressive Disorder Using Reasoning-Based Large Language Models: Model Development and Validation Study
Source: JMIR Ment Health. 2026 Jan 23;13:e83352. doi: 10.2196/83352 (PMC12829737; doi:10.2196/83352)
Supplement: Multimedia Appendix 5 [file mental-v13-e83352-s005.docx]

Mutimedia Appendix 5. Predictive Performance of Machine Learning Models for 12-Week Remission Classification

| **Data set** | **Models** | **Balanced Accuracy**  **(95% CI)** | **Sensitivity**  **(95% CI)** | **Specificity**  **(95% CI)** | **PPV**  **(95% CI)** | **NPV**  **(95% CI)** |
| --- | --- | --- | --- | --- | --- | --- |
| 5-fold CV  (n=331) | LR | 0.6077  (0.5856-0.6298) | 0.3533  (0.3063-0.4004) | 0.8620  (0.8431-0.8810) | 0.5958  (0.5499-0.6417) | 0.7023  (0.6880-0.7166) |
|  | RF | 0.6689  (0.6565-0.6813) | 0.4325  (0.4121-0.4529) | 0.9053  (0.8955-0.9151) | 0.7301  (0.7071-0.7530) | 0.7384  (0.7301-0.7466) |
|  | XGB | 0.6820  (0.6696-0.6943) | 0.5458  (0.5184-0.5733) | 0.8181  (0.8053-0.8308) | 0.6364  (0.6193-0.6535) | 0.7608  (0.7510-0.7706) |
| Test  (n=59) | LR | 0.6133  (0.5596-0.6669) | 0.3455  (0.2749-0.4160) | 0.8811  (0.8318-0.9303) | 0.6461  (0.5232-0.7691) | 0.6936  (0.6610-0.7262) |
|  | RF | 0.7181  (0.6840-0.7523) | 0.5227  (0.4691-0.5764) | 0.9135  (0.8785-0.9486) | 0.7891  (0.7256-0.8525) | 0.7635  (0.7398-0.7871) |
|  | XGB | 0.7371  (0.7002-0.7740) | 0.6364  (0.6021-0.6706) | 0.8378  (0.7781-0.8976) | 0.7133  (0.6342-0.7923) | 0.7940  (0.7732-0.8148) |
| **Abbreviations**: PPV, positive predictive value; NPV, negative predictive value; CV, cross-validation; LR, logistic regression; RF, random forest; XGB, XGBoost; CI, confidence interval.  ***Note***: This table summarizes the classification performance of machine learning models trained on the numerically coded dataset, including logistic regression, random forest, and XGBoost. Each model was evaluated using repeated 10×5-fold cross-validation and a held-out test set. Metrics include balanced accuracy, sensitivity, specificity, positive predictive value (PPV), and negative predictive value (NPV), with 95% bootstrap confidence intervals in parentheses. | | | | | | |
