# Supplementary material for: Prediction of 12-Week Remission in Patients With Depressive Disorder Using Reasoning-Based Large Language Models: Model Development and Validation Study
Source: JMIR Ment Health. 2026 Jan 23;13:e83352. doi: 10.2196/83352 (PMC12829737; doi:10.2196/83352)
Supplement: Multimedia Appendix 7 [file mental-v13-e83352-s007.docx]

Multimedia Appendix 7. Representative Remission (“No”) Case Generated by the RoD Prompting Strategy


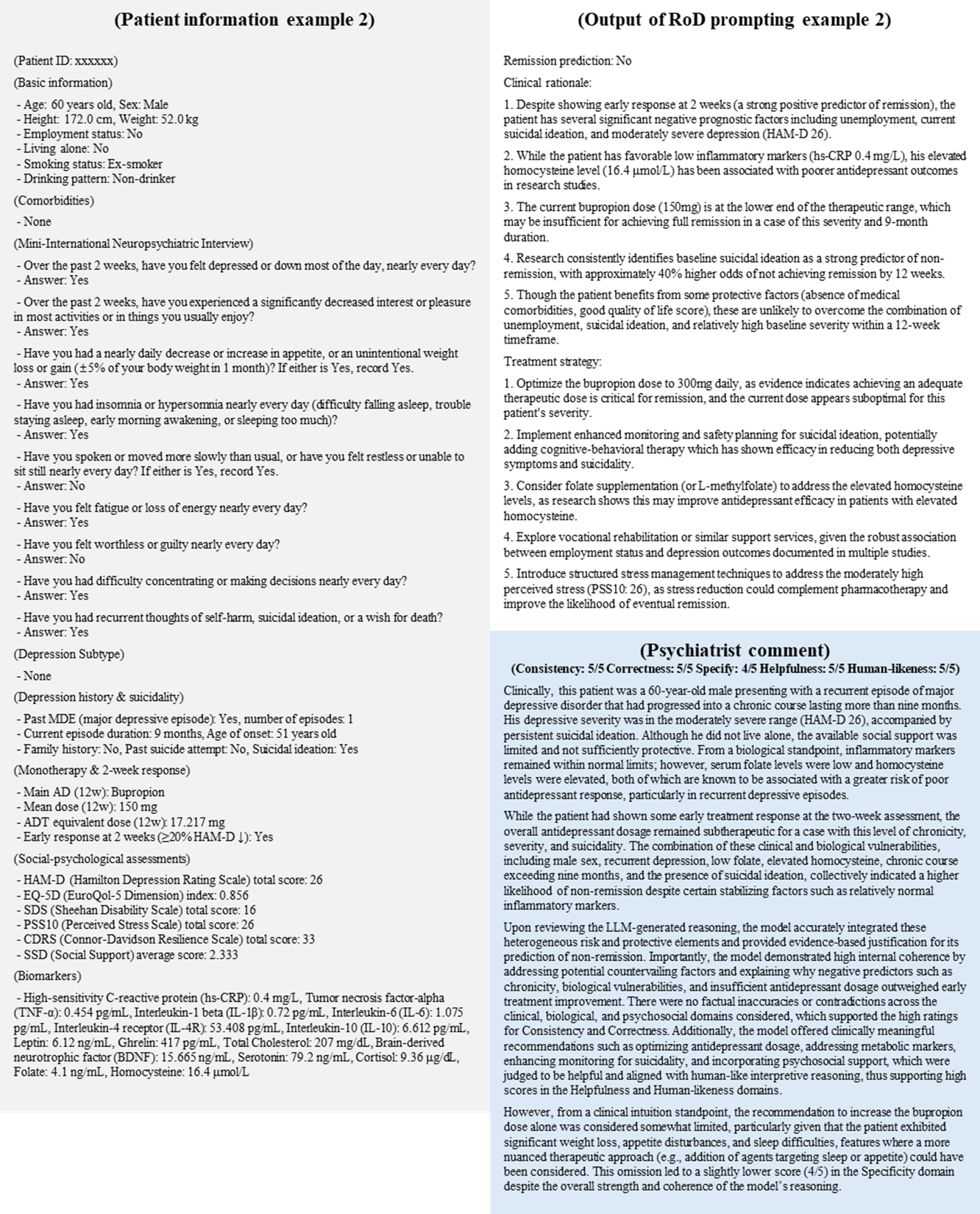


**Abbreviations**: RoD, referencing of deep research.

***Note***: This figure illustrates an example of a remission prediction (“No”) case produced by the RoD prompting strategy. The output includes the model’s five-sentence reasoning, the psychiatrist’s qualitative evaluation and commentary.
